# Comparative metagenomic analysis of gut microbiomes in Yunnan ponies and Dutch warmblood horses

**DOI:** 10.3389/fmicb.2026.1807081

**Published:** 2026-06-05

**Authors:** Jinlong Cha, Jieyan Yang, Zifang Zhang, Lindong Qian, Fangxiao Yang, Shiyou Li, Ju Li, Zonghui Jian, Wenjie Cheng

**Affiliations:** 1 Yunnan Vocational College of Agriculture, Kunming, China; 2 College of Veterinary Medicine, Yunnan Agricultural University, Kunming, China; 3 First People’s Hospital of Kunming City, Kunming, China

**Keywords:** antibiotic resistance genes, functional divergence, gut microbiota, metagenomics, Yunnan pony

## Abstract

**Introduction:**

The Yunnan pony is an officially protected pony breed in China. However, its gut microbiome characteristics remain largely unexplored. This study aimed to compare the gut microbiome and antibiotic resistance genes (ARGs) profiles between Yunnan ponies and Dutch warmblood horses.

**Methodology:**

A total of 14 fresh fecal samples were collected from Yunnan ponies and Dutch warmblood horses. Metagenomic sequencing was employed to comprehensively analyze and compare the gut microbial composition, function, and ARGs profiles between the two breeds.

**Results:**

The results showed no significant differences between the two breeds in core phylum composition or overall microbial diversity. A total of 146 bacterial genera were identified with significant differences at the genus level. Functional analysis revealed that the gut microbiota of Yunnan ponies was significantly enriched in pathways related to carbohydrate metabolism and pectin degradation, which are involved in basic energy acquisition. In contrast, Dutch warmblood horses were more enriched in host immune interaction pathways such as Toll-like receptor signaling. Analysis of ARGs indicated that while there was no difference in the overall diversity of ARGs between the two groups. Their association networks with specific bacterial hosts were markedly distinct, and the dominant ARG subtypes differed.

**Discussion:**

This study provides a descriptive characterization of the gut microbiome of Yunnan ponies, offering baseline data for future research on the conservation of this genetic resource and its health management in breeding.

## 1 Introduction

The horse is a typical hindgut-fermenting herbivore (Wunderlich et al., 2025). It hosts a complex and vast intestinal microbial community composed of bacteria, archaea, fungi, and viruses, which establishes a closely interactive symbiotic relationship with the host (Loublier et al., 2025; Zhu et al., 2025). The intestinal microbial community of horses secretes a variety of hydrolytic enzymes capable of degrading dietary fiber into volatile fatty acids, providing up to 70% of the host’s maintenance energy (Wunderlich et al., 2023). Beyond nutrient provisioning, the gut microbiota extensively participates in metabolic health, immune regulation, and the maintenance of intestinal barrier integrity (Barton et al., 2018). Specific microbial compositions are closely associated with the host’s digestive efficiency, immune homeostasis, and overall health status (Kondapalli et al., 2025). Therefore, the structural and functional homeostasis of the microbial community serves as a crucial foundation for equine health, disease resistance (Chaucheyras-Durand et al., 2022). In-depth analysis of the equine gut microbiome provides a scientific basis for maintaining animal health through nutritional strategies or probiotic interventions.

The Chinese pony is a valuable genetic resource characterized by high genetic diversity and a unique evolutionary history. In contrast to the European pony lineages, the Chinese pony retains ancient paternal haplotypes and genomic features (Li X. et al., 2025). The Yunnan pony is one of the five constituent breeds within the Chinese pony genetic resources (Han et al., 2025). Characterized by its small stature, gentle temperament, remarkable endurance, and unique phenotype adapted to mountainous environments, this breed was listed in the National Protected Breeds of Livestock and Poultry Genetic Resources in 2021 (Li Z. et al., 2025). Due to the development of the transportation industry and the advancement of modern agriculture, pony breeds are facing severe challenges, including the degradation of genetic resources and a gradual decline in population size and quality (Orlando and Librado, 2019). Long-term geographical isolation has preserved distinct Y-chromosome diversity between indigenous horse breeds in southwestern China, represented by the Yunnan pony, and European breeds as well as other local breeds within China (Liu et al., 2020). This distinct genetic background, coupled with physiological and metabolic traits adapted to mountainous environments and specific rearing practices, may collectively shape its distinct gut microbial ecosystem (Cao et al., 2023; Lan et al., 2024). Current research on the equine gut microbiome predominantly focuses on racehorses and sport horses. Although some studies have investigated pony breeds, the gut microbial composition and function of the Yunnan pony-as an original Chinese horse breed-remain poorly characterized (Mach et al., 2022; Park et al., 2024). Therefore, characterizing its gut microbiota will help enrich the microbial gene resource library of equine species and describe the breed-specific features of its gut microecology.

The functional potential encoded by carbohydrate-active enzymes (CAZy) and antibiotic resistance genes (ARGs) is of particular importance in equine gut microbiomes (Mitchell et al., 2021; He et al., 2026). CAZy families govern the breakdown of complex plant polysaccharides-the primary energy substrates for hindgut fermenters-and their differential enrichment between breeds reveals breed-specific capabilities in fiber utilization (Flint et al., 2012). Understanding these enzymatic profiles can inform nutritional management for local breeds such as the Yunnan pony. On the other hand, ARGs represent a reservoir of resistance traits whose distribution and host association patterns are shaped by microbial community structure and environmental factors (Mitchell et al., 2023). Even in the absence of recent antibiotic exposure, the presence of transferable ARGs in core commensal bacteria raises questions about the ecological and evolutionary drivers of resistance gene maintenance (Knoppel et al., 2017). Thus, characterizing CAZy and ARG profiles not only expands our knowledge of functional diversity in equine microbiomes but also provides baseline data for health monitoring and risk assessment.

To address these knowledge gaps, the present study employs metagenomic sequencing to profile the gut microbiomes of Yunnan ponies (*n* = 9) and Dutch Warmblood horses (*n* = 5). This study aims to compare the taxonomic composition, diversity, and functional potential (including CAZy enzymes and KEGG pathways) of the gut microbiota between an indigenous breed (Yunnan pony) and a sport horse breed (Dutch Warmblood); and characterize the antibiotic resistance gene (ARG) profiles of these two horse breeds, including ARG diversity, abundance, and their potential bacterial hosts. This study will provide foundational microbiota-level data to support the conservation and health management of indigenous horse genetic resources.

## 2 Materials and methods

### 2.1 Sample collection

A total of 14 fresh equine fecal samples were collected for this study. All horses originated from a single farm in Kunming, Yunnan Province, and included Yunnan ponies (*n* = 9) and Dutch Warmblood horses (*n* = 5). The Dutch Warmblood horses were imported from Belgium at the age of 2 years. The horses were clinically healthy and had not received any antibiotics or anthelmintics for at least 2 months prior to sampling. Detailed information for all horses is provided in Supplementary Table 1. Each horse was housed in an individual stall. Before sampling, all horses had been stably maintained in their respective housing environment for at least 6 months, under uniform management and fed an identical diet. Approximately 10 g of feces was collected from the inner portion of the freshly deposited sample immediately after defecation.

### 2.2 Microbial genomic DNA extraction and metagenomic sequencing

Total genomic DNA of the microbial community was extracted using the FastPure Stool DNA Isolation Kit (Magnetic bead) (MJYH, Shanghai, China). DNA integrity and quality were assessed by 1% agarose gel electrophoresis. The DNA was fragmented using a Covaris M220 (Genomics Co., Ltd., China), and fragments of approximately 350 bp were selected for paired-end (PE) library construction. Libraries were prepared using the NEXTFLEX Rapid DNA-Seq Kit (Bioo Scientific, United States). Libraries passing quality control were subjected to PE150 sequencing on the Illumina NovaSeq 6000 platform. Each sample was sequenced to a target depth of 6 Gb (approximately 20 million paired-end reads). Libraries from all 14 samples were pooled in equimolar ratios and loaded onto one lane of the flow cell for sequencing.

### 2.3 Sequencing data processing and assembly

Data quality control was performed using fastp v0.20.0 (Chen et al., 2018) to trim adapters and remove reads < 50 bp or with average quality < Q20. BWA v0.7.17 (Li and Durbin, 2009) was used to remove host-derived reads. Qualified reads were assembled using MEGAHIT v1.1.2 (Li et al., 2015); contigs < 300 bp were discarded. Open reading frames (ORFs) within the assembled contigs were predicted using Prodigal v2.6.3 (Hyatt et al., 2010); ORFs < 100 bp were filtered. A non-redundant gene set was constructed using CD-HIT v4.7 (Fu et al., 2012) with 90% identity and 90% coverage. Gene abundance was calculated by aligning reads to the non-redundant gene set using SOAPaligner v2.21 (Li et al., 2008) at 95% identity.

### 2.4 Taxonomic composition analysis

Taxonomic annotation was obtained by aligning the non-redundant gene set to the NR database using Diamond v2.0.13 (Buchfink et al., 2015) with e-value ≤ 1e-5. The relative abundance of each taxon was calculated as the number of reads annotated to that taxon divided by the total number of annotated reads in the sample. The total numbers of bacterial phyla, genera, and species identified across all samples, as well as the mean relative abundances of the dominant taxa, were reported.

### 2.5 Diversity analysis

Alpha diversity of bacterial communities was estimated using the ACE and Shannon indices. Differences in alpha diversity between Yunnan ponies and Dutch Warmblood horses were tested using the Wilcoxon rank-sum test. Beta diversity was assessed based on Bray-Curtis distances via principal component analysis (PCA) and principal coordinates analysis (PCoA). PERMANOVA (with 999 permutations) was used to test for significant differences in community structure between the two groups.

### 2.6 Differential analysis of bacterial genera

Differential abundance of bacterial genera between Yunnan ponies and Dutch Warmblood horses was tested using the Wilcoxon rank-sum test as implemented in STAMP v2.1.1.0 (Parks et al., 2014). Genera with uncorrected *P* < 0.05 were considered potentially different. Given the exploratory nature of the study and the limited sample size, no multiple test correction was applied, and results should be interpreted as hypothesis-generating.

### 2.7 Functional enrichment analysis

Functional annotation was performed by aligning the non-redundant gene set to the KEGG database (release 20,230,830) (Kanehisa et al., 2025) using Diamond v2.0.13 (Buchfink et al., 2015) with e-value ≤ 1e-5, and the abundances of functional categories were calculated. KEGG pathways enrichment analysis was performed using the Generalized Reporter Score-based Analysis (GRSA) method implemented in the R package ReporterScore (version 0.1.8) (Peng et al., 2024). Input data consisted of normalized relative abundance values of KO features. For each pathway, KO *p*-values (Wilcoxon rank-sum test) were Z-transformed and aggregated into a reporter score. Pathways with | reporter score| ≥ 1.65 (α = 0.05) were considered significant. Default parameters included 1,000 permutation iterations and the built-in KEGG database. No multiple test correction (neither at the KO level nor at the pathway level) was applied.

Carbohydrate-active enzyme (CAZy) family annotation was performed by aligning the amino acid sequences of the non-redundant gene set to the CAZy database (v8) (Drula et al., 2022) using HMMER v3.1b2 (Eddy, 2011) with e-value ≤ 1e-5, and the abundances of enzyme classes were calculated. The abundance of each CAZy family was compared between the two breeds using the Wilcoxon rank-sum test (STAMP v2.1.1.0), with uncorrected *P* < 0.05 considered suggestive of differential abundance.

### 2.8 Antibiotic resistance gene (ARG) analysis

Antibiotic resistance annotation was obtained by aligning the amino acid sequences of the non-redundant gene set to the CARD database (v3.0.9) (Alcock et al., 2023) using Diamond v2.0.13 (Buchfink et al., 2015) with e-value ≤ 1e-5, and the corresponding functional annotations and abundances were obtained. The relative abundance of each ARG subtype and ARG class was calculated. Alpha diversity of ARGs (ACE and Shannon indices) and beta diversity (Bray-Curtis based PCA/PCoA) were compared between the two breeds using the same methods as described in section 2.5. Differential abundance of ARG subtypes was tested using the Wilcoxon rank-sum test (STAMP v2.1.1.0) with uncorrected *P* < 0.05, and no multiple test correction was applied. Given the exploratory nature and limited sample size, all reported *P*-values from the Wilcoxon rank-sum tests were not corrected for multiple comparisons, and the results should be interpreted as hypothesis-generating. Data analysis was conducted on the free online Majorbio Cloud Platform^1^ (Han et al., 2024).

### 2.9 Correlation network analysis

Co-occurrence networks between bacterial genera and ARG subtypes were constructed based on Spearman correlation coefficients (| *r*| > 0.7, *P* < 0.05). Networks were visualized using Gephi v0.10.1 (Bastian et al., 2009).

## 3 Results

### 3.1 Sequencing and assembly outcomes

Metagenomic sequencing of 14 fecal samples generated 40,797,792–49,069,872 raw reads per sample, with raw bases ranging from 6,160,466,592 to 7,409,550,672 bp per sample. After quality control, clean reads per sample ranged from 40,410,686 to 48,435,582, and clean bases ranged from 6,089,577,010 to 7,296,557,290 bp, representing 97.2–99.2% of raw reads. The average number of clean reads per sample was approximately 44.8 million, with a standard deviation of 2.5 million and a coefficient of variation of 5.6%, indicating consistent sequencing output across samples. Assembly yielded 514,347–755,692 contigs per sample (N50: 507–965 bp). The number of predicted ORFs per sample ranged from 598,250 to 983,237. After redundancy removal, a non-redundant gene catalog consisting of 5,856,105 genes was constructed, with an average length of 550 bp (Supplementary Table 2). The sequencing depth and assembly quality of this dataset are sufficient for robust downstream analyses of microbial community composition and functional potential.

### 3.2 Composition of the horse gut microbiota

**FIGURE 1:**
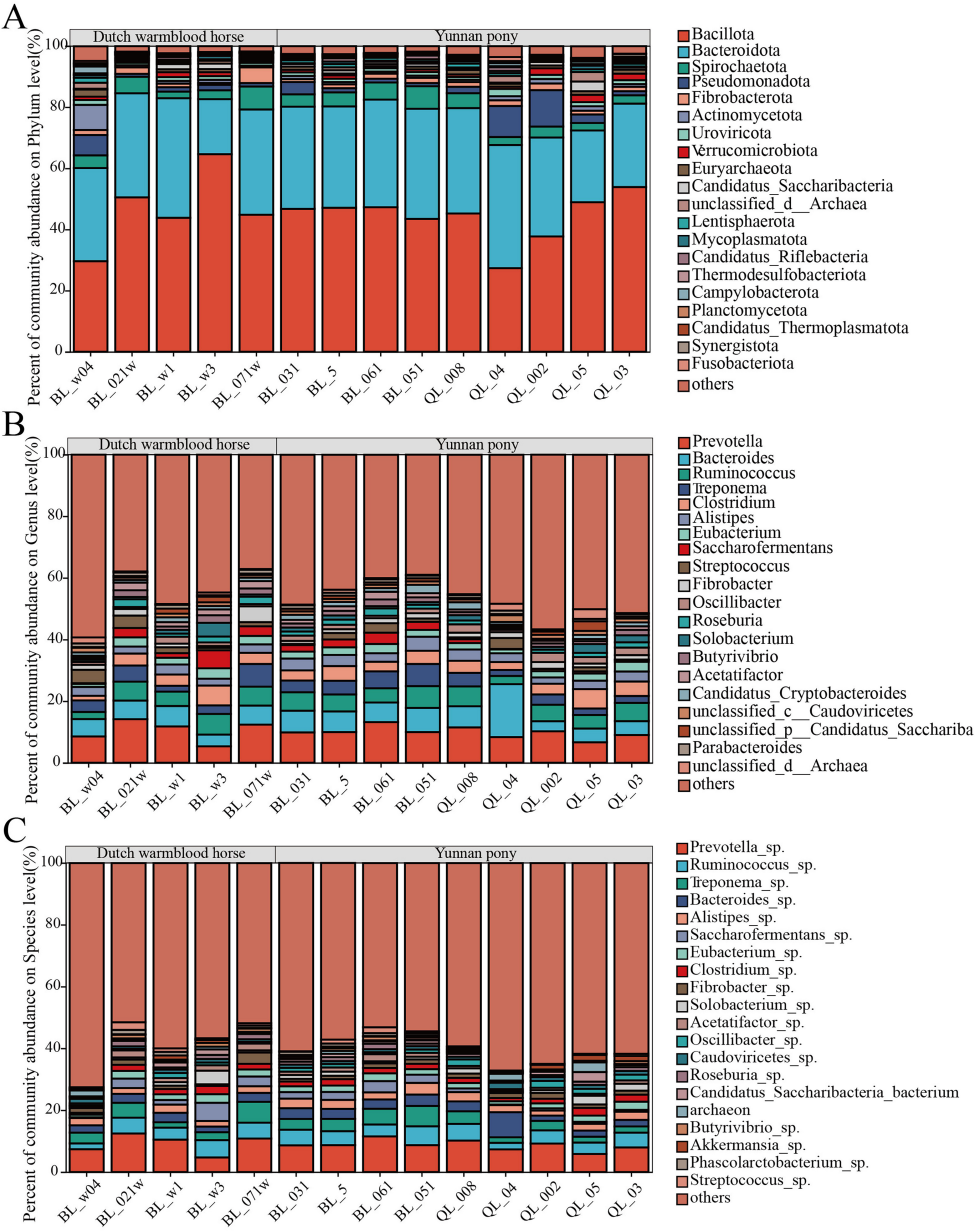
Microbiome composition of 14 horses at the phylum **(A)**, genus **(B)**, and species **(C)** levels. “Others” represents the combined abundance of all taxa not within the top 20 most abundant. “Unclassified” indicates sequences that could not be assigned to a known taxon at that level based on the NCBI-NR database.

A total of 230 bacterial phyla, 5,581 genera, and 39,440 species were identified across all 14 samples. At the phylum level, Bacillota and Bacteroidota were the dominant phyla in the equine gut, with mean relative abundances of 45 and 32%, respectively, across all samples (Figure 1A). At the genus level, the five most abundant genera across all samples were *Prevotella* (9%), *Bacteroides* (6%), *Ruminococcus* (5%), *Treponema* (4%), and *Clostridium* (4%) (Figure 1B). At the species level, the five most abundant species were *Prevotella* sp. (8%), *Ruminococcus* sp. (4%), *Treponema* sp. (3%), *Bacteroides* sp. (3%), and *Alistipes* sp. (2%) (Figure 1C).

### 3.3 Diversity analysis of the horse gut microbiota

**FIGURE 2:**
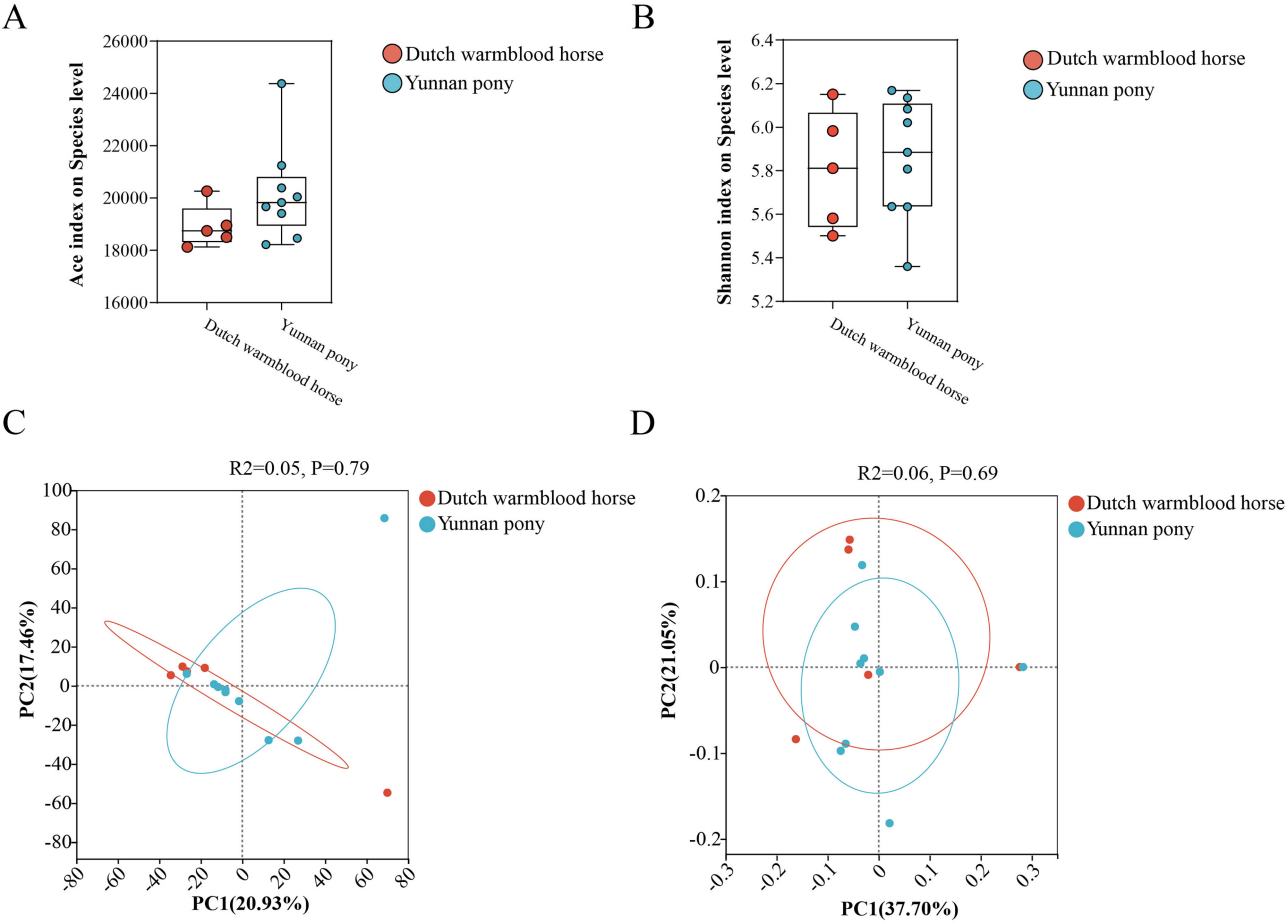
Diversity analysis of gut microbiota in different horse breeds. **(A)** ACE index. **(B)** Shannon index. **(C)** Principal Component Analysis (PCA) of beta diversity in gut microbiota across different breeds based on Bray-Curtis distance. **(D)** Principal Coordinate Analysis (PCoA) of beta diversity in gut microbiota across different breeds based on Bray-Curtis distance. Uncorrected *P*-values from Wilcoxon rank-sum tests are reported. Ellipses represent 95% confidence intervals for each breed. PERMANOVA analysis showed that the breed factor had no significant effect on microbial β-diversity.

Alpha diversity analysis revealed no significant differences between Yunnan ponies and Dutch Warmblood horses in either the ACE index (Wilcoxon rank-sum test, *P* = 0.59) or the Shannon index (*P* = 0.42) (Figures 2A,B). Beta diversity analysis using Bray-Curtis distances showed that breed did not influence the overall structure of the equine gut microbiota (PERMANOVA, *P* > 0.05) (Figures 2C,D).

### 3.4 Screening of differential microbiota

**FIGURE 3:**
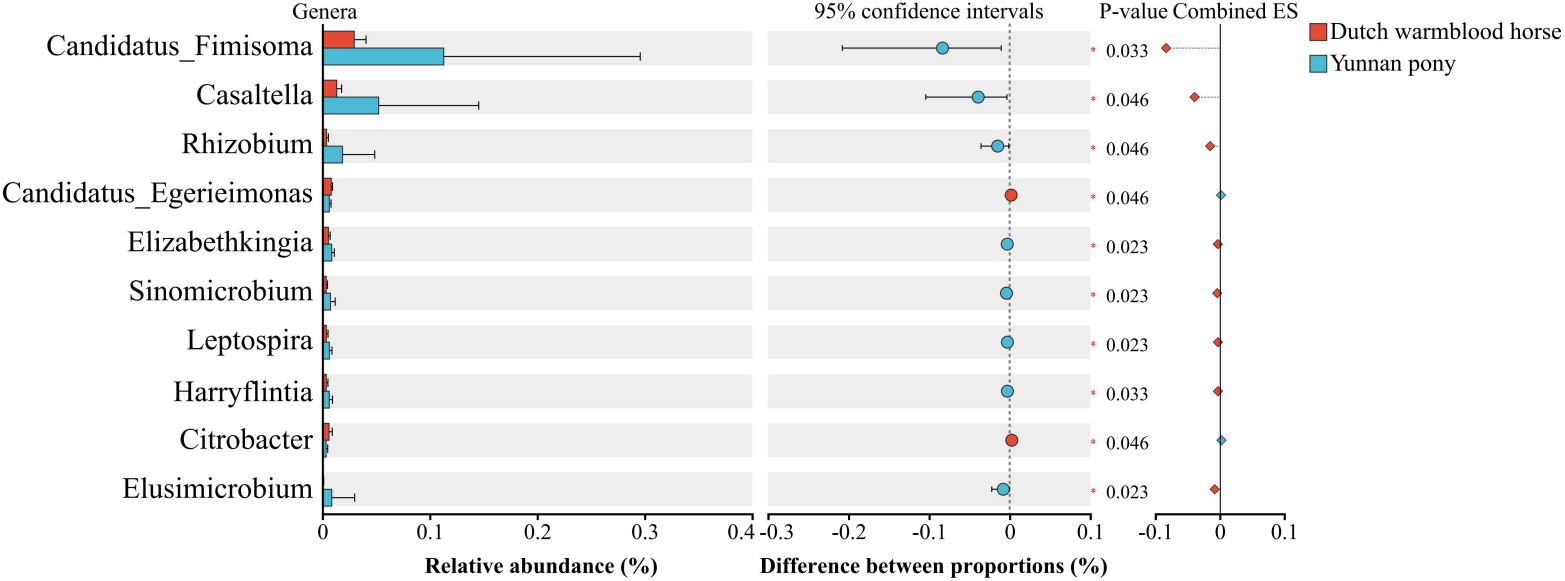
Top 10 genera ranked by relative abundance among the differentially abundant bacteria. Genera are ranked by median relative abundance. Error bars represent standard error of the mean. Combined ES: Combined Effect Size (magnitude and direction of difference between groups). Full statistical results are provided in Supplementary Table 2. Uncorrected *P*-values from Wilcoxon rank-sum tests are reported.

Differential analysis of the microbial communities between the two breeds was performed using the Wilcoxon rank-sum test. The results revealed suggestive differences (uncorrected *P* < 0.05) in the relative abundances of 146 bacterial genera between Yunnan ponies and Dutch warmblood horses. Specifically, 126 genera, including *Candidatus* Fimisoma, *Casaltella*, *Rhizobium* and *Elusimicrobium*, were higher in the Yunnan pony group (*P* < 0.05), while 20 genera, such as *Candidatus* Egerieimonas, were lower in the Yunnan pony group (*P* < 0.05) (Figure 3 and Supplementary Table 3).

### 3.5 Differential functional enrichment of gut microbiota

**FIGURE 4:**
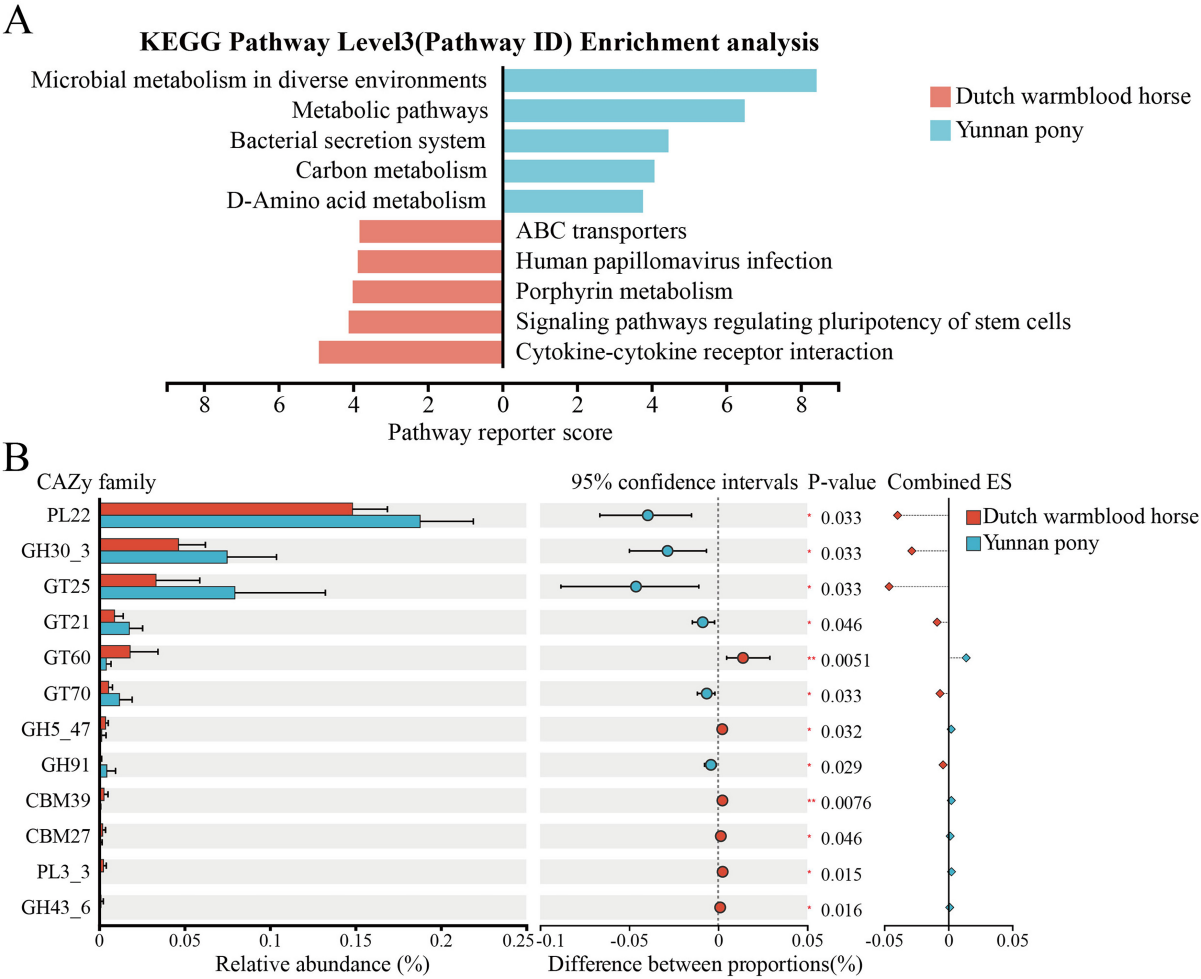
Comparative analysis of microbial metabolic functions and carbohydrate-active enzyme profiles. **(A)** Top 10 enriched KEGG pathways. Pathways with | ReporterScore| ≥ 1.65 are significantly enriched. ReporterScore: enrichment score reflecting the significance and direction of pathway activation. **(B)** Differentially abundant CAZy families. Combined ES: Combined Effect Size (magnitude and direction of difference between groups). PL, polysaccharide lyase; GH, glycoside hydrolase; GT, glycosyltransferase; CBM, carbohydrate-binding module. Uncorrected *P*-values (Wilcoxon rank-sum test) are reported for CAZy family comparisons. Left panel: mean relative abundance ± standard error of the mean (error bars). Middle panel: 95% confidence intervals for the difference in means (Yunnan pony minus Dutch Warmblood).

To systematically investigate the overall functional differences between the gut microbial communities of Dutch warmblood horses and Yunnan ponies, pathway-level enrichment analysis was performed using the Reporter Score algorithm. A total of 471 KEGG pathways were detected in the horse gut microbiome. Among them, 155 differentially abundant KEGG pathways were identified (absolute reporter score ≥ 1.65). KEGG functional enrichment analysis revealed distinct functional profiles between the two breeds. In the Yunnan pony group, the enriched pathways were primarily core metabolic processes (carbon metabolism, amino acid biosynthesis, oxidative phosphorylation) and biofilm formation. In the Dutch warmblood horse group, the enriched pathways were mainly host immune interaction and signaling pathways (Cytokine-cytokine receptor interaction, Toll-like receptor, PI3K-Akt, MAPK) (Figure 4A and Supplementary Table 4).

To further elucidate the functional enrichment in basic energy metabolism pathways in Yunnan ponies, the Carbohydrate-Active Enzymes (CAZy) profiles of the microbial communities were analyzed. A total of 607 CAZy families were identified across all samples. The results showed differential enrichment of CAZy profiles between Yunnan ponies and Dutch warmblood horses. Among the differentially abundant families, those with known roles in plant cell wall degradation were examined based on CAZy database annotations (Drula et al., 2022). In the gut microbiota of Yunnan ponies, the gene abundances of pectin lyase (PL22) (involved in pectin degradation), certain glycoside hydrolases (e.g., GH30_3), and glycosyltransferases (e.g., GT21, GT25, GT70), were potentially higher than in Dutch warmblood horses (uncorrected *P* < 0.05). Additionally, an enzyme family involved in glycogen/starch degradation (GH91) was also a candidate for enrichment in the Yunnan pony group. In the Dutch warmblood horse group, the enriched families included GH5_47 and GH43_6 (both involved in hemicellulose/xylan/arabinan degradation), carbohydrate-binding modules CBM27 and CBM39, as well as PL3_3 and GT60 (Figure 4B).

### 3.6 Analysis of antibiotic resistance genes (ARGs)

**FIGURE 5:**
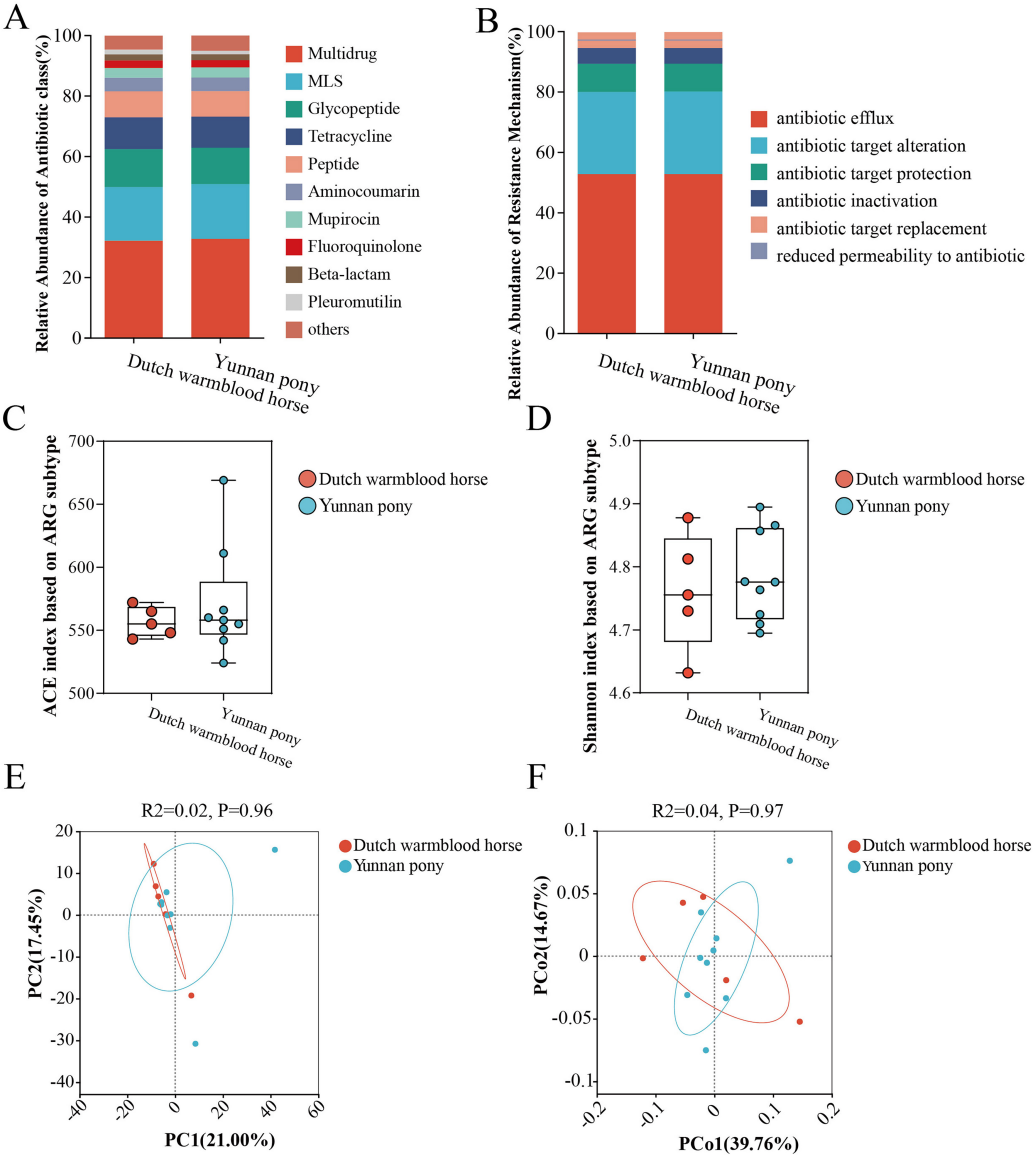
Profiles of antibiotic resistance genes (ARGs) in equine gut microbiota. **(A)** Relative abundance of the top-10 antibiotic classes. “Others” represents all classes outside the top-10. **(B)** Relative abundance of resistance mechanisms. **(C)** ACE index. **(D)** Shannon index. **(E)** Principal Component Analysis (PCA) of ARGs between the two groups. **(F)** Principal Coordinate Analysis (PCoA) of ARGs between the two groups. Uncorrected *P*-values from Wilcoxon rank-sum tests are reported. PERMANOVA showed no significant separation (*P* > 0.05). All analyses were performed on ARG relative abundances. MLS, macrolide-lincosamide-streptogramin.

A total of 891 ARG subtypes (across 21 classes) were detected in the 14 samples (Supplementary Table 5), including those conferring resistance to acridine dye, aminocoumarin, benzalkonium chloride, bicyclomycin, carbapenem, and cephalosporin. The top five ARG classes with the highest abundance in both Yunnan ponies and Dutch warmblood horses were Multidrug, MLS, Glycopeptide, Tetracycline, and Peptide (Figure 5A). The most abundant antibiotic resistance mechanisms in both Yunnan ponies and Dutch warmblood horses were antibiotic efflux, antibiotic inactivation, and antibiotic target alteration (Figure 5B). There was no difference in the alpha diversity of ARGs between Yunnan ponies and Dutch warmblood horses (Figures 5C,D), and the beta diversity of ARG composition also showed no significant difference between the two groups (*P* > 0.05) (Figures 5E,F). Compared with the Dutch Warmblood group, the potentially increased ARGs in the Yunnan Pony group included *CepS*, *aphA15*, *ErmR*, *vanRA*, *Erm(49)*, and *SRT-2* (uncorrected *P* < 0.05). Some ARGs were potentially decreased in the Yunnan Pony group, including *tetA(46)*, *catB8*, *ADC-16*, *AAC(6’)-Iy*, *hmrM*, *CfxA6*, and *efpA* (uncorrected *P* < 0.05) (Supplementary Table 6).

### 3.7 Correlation network analysis

**FIGURE 6:**
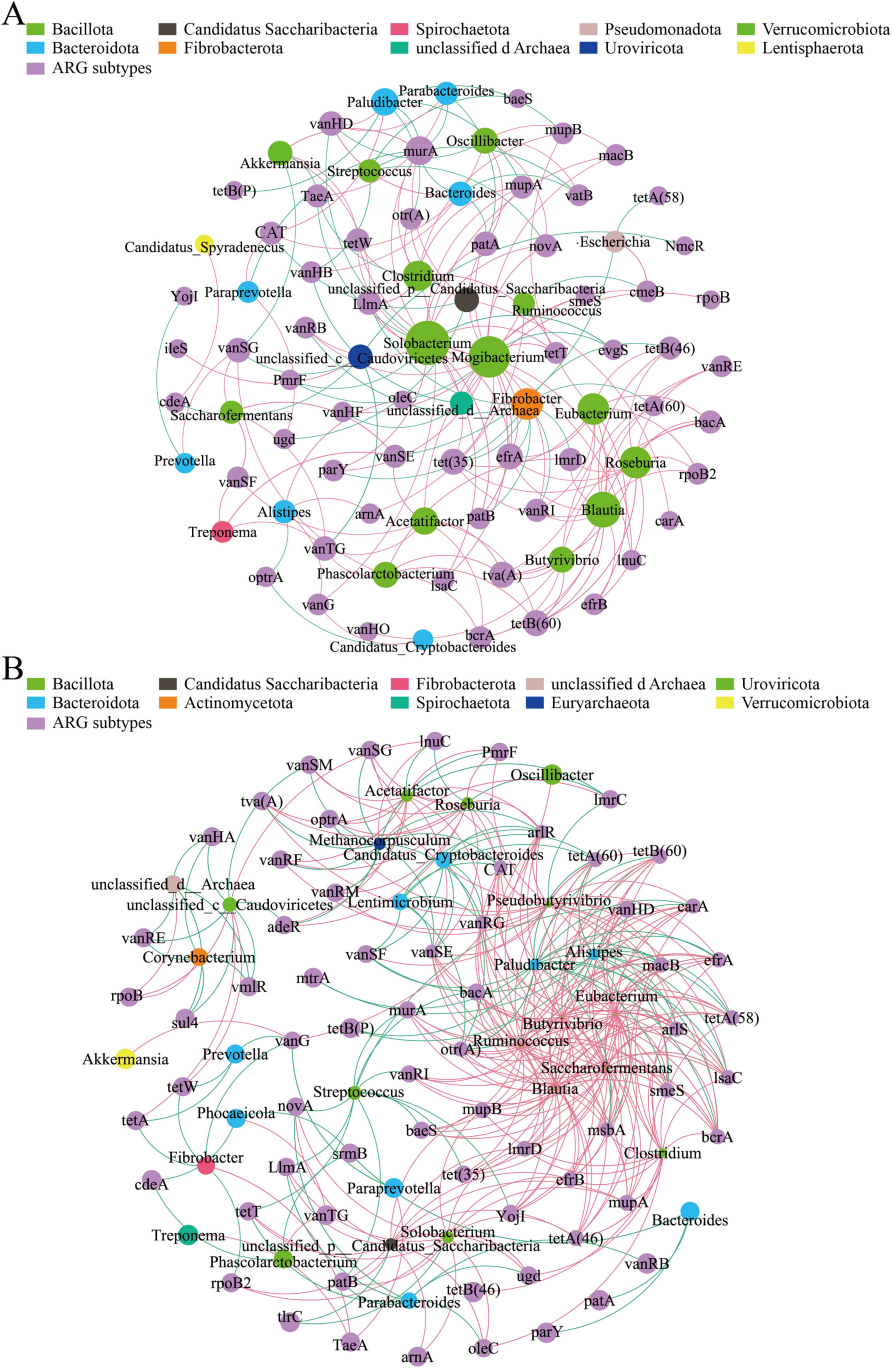
Co-occurrence network analysis between ARG subtypes and microbial taxa. **(A)** Yunnan ponies. **(B)** Dutch warmblood horses. Edges indicate significant correlations (*r* > 0.7, *P* < 0.05). Only nodes with at least one connection are shown. All pairwise associations (including correlation coefficients and *P*-values) are listed in Supplementary Table 6.

The co-occurrence network between microbial genera and ARG subtypes is shown in Figure 6. The results revealed differences in the composition of dominant bacterial genera acting as potential hosts for ARGs between the two groups. In the Yunnan pony group, 57 ARG subtypes were potentially hosted by 28 bacterial genera. *Solobacterium*, *Mogibacterium*, and *Blautia* were the primary genera associated with ARGs, serving as potential hosts for 19, 17, and 13 ARG subtypes, respectively (Figure 6A). In the Dutch warmblood horse group, 66 ARG subtypes were potentially hosted by 30 bacterial genera. *Ruminococcus*, *Butyrivibrio*, *Eubacterium*, *Blautia*, and *Saccharofermentans* showed significant positive correlations with multiple ARG subtypes, each serving as a potential host for 28 ARG subtypes (Figure 6B). Genera unique to Yunnan ponies (3 genera) were *Candidatus* Spyradenecus (associated with 1 ARG subtype), *Escherichia* (3 ARG subtypes), and *Mogibacterium* (17 ARG subtypes). Genera unique to Dutch warmblood horses (5 genera) were *Corynebacterium* (5 ARG subtypes), *Lentimicrobium* (7 ARG subtypes), *Methanocorpusculum* (14 ARG subtypes), *Phocaeicola* (3 ARG subtypes), and *Pseudobutyrivibrio* (22 ARG subtypes) The remaining 25 genera were shared between the two groups, and the complete list of shared genera is provided in Supplementary Figure 1A and Supplementary Table 7. Furthermore, from the perspective of specific ARG subtypes, the genes with the highest dissemination potential also differed between the two groups. In the Yunnan pony group, *Chlamydia trachomatis intrinsic murA conferring resistance to fosfomycin*, *efrA, tet(35)*, *tetB(60)*, *tva(A)* were carried by 8, 6, 6, 6, and 6 bacterial genera, respectively (Figure 6A). Among these, *tetB(60)* and *tva(A)* had no detectable bacterial hosts in Dutch warmblood horses, while *murA* and *tet(35)* were present at comparable levels (6 genera each) and *efrA* was even more abundant (9 genera). In the Dutch warmblood horse group, *arlR*, *vanRG*, and *vanSE* were each carried by 11 bacterial genera (Figure 6B). *arlR* and *vanRG* showed no association with any bacterial genus in Yunnan ponies, whereas *vanSE* was associated with only 4 genera. Thus, the dominant ARGs in each group show different patterns of host prevalence across the two breeds. All pairwise associations are listed in Supplementary Table 7. 34 ARG subtypes were shared between the two groups, 23 were unique to Yunnan ponies, and 32 were unique to Dutch Warmblood horses (Supplementary Figure 1B). These observations indicate that the two breeds differ not only in their dominant host bacterial genera but also in the specific ARG subtypes and their associated bacterial hosts.

## 4 Discussion

This study is the first to characterize the gut microbiome of the Yunnan pony through metagenomics. Although the core microbiota and overall diversity of the Yunnan pony and Dutch warmblood horses were similar, suggestive differences were observed in genus-level composition, functional potential, and ARGs host networks. The study found that Bacillota and Bacteroidota were the dominant bacterial phyla in both Yunnan ponies and Dutch warmblood horses, which aligns with previous equine research (Li et al., 2023; Carrillo Heredero et al., 2024). However, at the genus level, 146 bacterial genera showed suggestive differences in relative abundance between the Yunnan pony and Dutch warmblood horse groups. These findings suggest that while core fermentation functions remain stable, the microbial community exhibits high plasticity at finer taxonomic levels across different horse breeds under diverse management conditions (Li et al., 2023; Yao et al., 2025). In this study, no differences were observed in the alpha and beta diversity of gut bacteria between the two breeds. Concurrently, a study comparing gut fungi between two horse breeds (Dutch Warmblood and Mongolian horse) also reported no significant differences in alpha or beta diversity (Lan et al., 2024). This finding, together with our bacterial diversity results, is consistent with the idea that horses, as hindgut fermenters, possess a highly conserved core microbial functionality at higher taxonomic levels (Chaucheyras-Durand et al., 2022).

We found that the gut microbial functions of Yunnan ponies were suggestively enriched in core metabolic pathways, including carbon metabolism, amino acid biosynthesis, and oxidative phosphorylation, all of which are central to energy production and biosynthesis. In contrast, the gut microbiota of Dutch warmblood horses was suggestively enriched in immune-related signaling pathways, such as cytokine-cytokine receptor interaction, Toll-like receptor (TLR) signaling, PI3K-Akt, and MAPK pathways. This functional divergence reflects the difference in gut microbiota functional potential between the two horse breeds (Yao et al., 2025). Yunnan ponies may enhance their fiber degradation and energy acquisition capabilities through their microbiome (Wunderlich et al., 2025). The functional characteristics of Dutch warmblood horses may be associated with their relatively refined management environment. TLRs are key molecules for the intestinal epithelium to recognize microbes and regulate immune homeostasis (Eislmayr et al., 2025). Studies have shown that factors such as diet can alter the gut microbiota, thereby influencing intestinal immune balance by modulating the expression of pattern recognition receptors like TLR and related cytokines (Kaur and Ali, 2022). Other research has also observed significant metabolic functional differences among different horse breeds (Qin et al., 2025). Therefore, the functional divergence revealed in this study may be influenced by multiple factors, including breed-specific backgrounds, rearing practices, and others. However, the contribution of these factors to the functional divergence cannot be determined in this study.

The suggestive enrichment of pectin lyase (PL22) in the Yunnan pony group suggests that their microbiome may possess a greater advantage in the disassembly of pectin. Pectin is an important component of plant cell walls, and its efficient degradation helps release encapsulated cellulose and hemicellulose, thereby promoting high-efficiency fermentation of fiber (Flint et al., 2012). The enrichment of certain glycosyl transferases (GT families) further points to differences in glycan biosynthesis between the two groups (Egelund et al., 2006; Wang et al., 2018). In contrast, the suggestively enriched enzymes in the Dutch warmblood horse group (such as GH5_47 and GH43_6) are more focused on the in-depth degradation of specific types of hemicellulose (e.g., xylan and arabinan) (Geng et al., 2022; Martins et al., 2022). This indicates that the two breeds differ in their enzyme profiles, which may reflect differences in their rearing environments prior to the 6-month acclimation period. Specifically, Yunnan ponies have traditionally been raised under free-range or semi-free-range conditions with coarse forage (high in fiber), whereas Dutch Warmblood horses typically originate from intensive breeding systems with more refined feeding (often including concentrates and managed pastures). Beyond differences in rearing environments, other factors may also contribute to the observed differences in gut microbiota and functional profiles. Host genetics have been shown to influence gut microbial composition in horses (Massacci et al., 2020). Maternal microbial transmission during early life critically shapes the initial gut colonization of foals (Husso et al., 2020). Additionally, transportation can transiently alter intestinal motility and microbial composition (Szemplinski et al., 2020; Raidal et al., 2025). In the current study, the fact that Dutch Warmblood horses were imported from Belgium at 2 years of age, while Yunnan ponies were born and raised at the local farm, may have contributed to differences in early-life microbial exposure and potentially shaped the observed functional and taxonomic profiles. Furthermore, weaning, age, and other management-related factors are known to modulate the equine gut microbiota (Garber et al., 2020). These early life differences in diet and husbandry could shape baseline microbial enzyme profiles, even after a period of uniform management.

Although there was no significant difference in the overall diversity and composition of ARGs between the two breeds, correlation network analysis revealed notable differences in the association patterns between ARGs and specific bacterial hosts. These differences reflect the distinct structures of the gut microbial communities in the two groups (Wang et al., 2023). In Dutch warmblood horses, ARGs were predominantly enriched in genera such as *Ruminococcus* and *Butyrivibrio*, which are generally regarded as core fiber-degrading bacteria (Dassa et al., 2014; Rabee et al., 2023). Additionally, ARGs were closely linked with bacteria like *Pseudobutyrivibrio*, a genus uniquely enriched in this breed, which also possesses crucial fermentation functions (Zhang et al., 2024). In Yunnan ponies, ARGs were more closely associated with *Mogibacterium* (a genus unique to this breed), as well as with shared fiber-degrading genera including *Roseburia* and *Eubacterium* (Louis and Flint, 2009; Mi et al., 2018; Mukherjee et al., 2020). Although Roseburia and Eubacterium were present in both groups, their patterns of ARG carriage differed. This finding suggests that the distribution and transmission potential of ARGs in the equine gut are highly dependent on their bacterial hosts, rather than being directly determined by horse breeds (Jian et al., 2025). At the same time, it indicates that different rearing environments (as described above) and management practices, by shaping distinct dominant bacterial communities, indirectly influence the host spectrum of ARGs and their potential transmission risks (Li et al., 2022). There is a clear difference in the distribution of transmissible ARG subtypes between Yunnan ponies and Dutch warmblood horses. However, it should be noted that none of the horses in either group had received antibiotic treatment within three months prior to sampling. Therefore, the observed differences in ARG distribution may be influenced by other factors, such as differences in rearing environments (prior to the study), historical antibiotic exposure, or environmental reservoirs of ARGs (Wang et al., 2023). The frequently prevalent *vanRG* and *vanSE* (vancomycin resistance-related genes) in the Dutch warmblood horse group are often associated with resistance to clinically common antibiotics (Bai et al., 2024). *MurA* is enriched in the Yunnan pony group and has been detected in *Staphylococcus aureus* isolated from Yunnan cheese (Sri Prabakusuma et al., 2022). As *S. aureus* is a common commensal of the gut and skin microbiota in horses and cows (Dimitracopoulos et al., 1977; Mahmmod et al., 2018; Kaspar et al., 2019; Wesolowska and Szczuka, 2025), this finding is not unexpected. Nevertheless, the presence of this ARG in a food-related bacterial species highlights the importance of including it in localized antibiotic resistance surveillance. The widespread presence of these ARG subtypes across multiple bacterial genera suggests that they may possess strong potential for horizontal gene transfer (Swain and Sahoo, 2025).

This study has several limitations. First, the sample size is relatively small (*n* = 14), which precluded a robust multivariate analysis incorporating age, sex, and husbandry-related variables as covariates. Although our preliminary checks did not reveal significant effects of age or sex on the main outcomes, a larger and more balanced cohort is required to definitively disentangle the contributions of breed, age, sex, and environmental factors. Future studies with expanded sample sizes and controlled covariate designs are needed to validate and extend our findings.

In conclusion, this study presents the first systematic characterization of the gut microbiome of Yunnan ponies. Differences were observed between Yunnan ponies and Dutch warmblood horses in terms of genus-level composition, metabolic functions, and the host networks of ARGs. Yunnan ponies demonstrated stronger potential for fiber degradation, whereas Dutch warmblood horses were enriched in immune interaction pathways. The transmission of ARGs was highly dependent on their host bacteria, and the prevalent ARG subtypes differed between the two groups. This research provides an important scientific foundation for the conservation and healthy breeding of Yunnan ponies as a valuable genetic resource.

## Data Availability

The data presented in the study are deposited in the NCBI repository, accession number PRJNA1420381.

## Footnotes

1
www.majorbio.com

## Supplementary material

Supplementary Figure 1 Venn diagrams showing shared and unique bacterial genera **(A)** and ARG subtypes **(B)** between Yunnan ponies and Dutch Warmblood horses.
