# Supplementary figures and images for: Comparative metagenomic analysis of gut microbiomes in Yunnan ponies and Dutch warmblood horses
Source: Front Microbiol. 2026 Jun 5;17:1807081. doi: 10.3389/fmicb.2026.1807081 (PMC13279432; doi:10.3389/fmicb.2026.1807081)

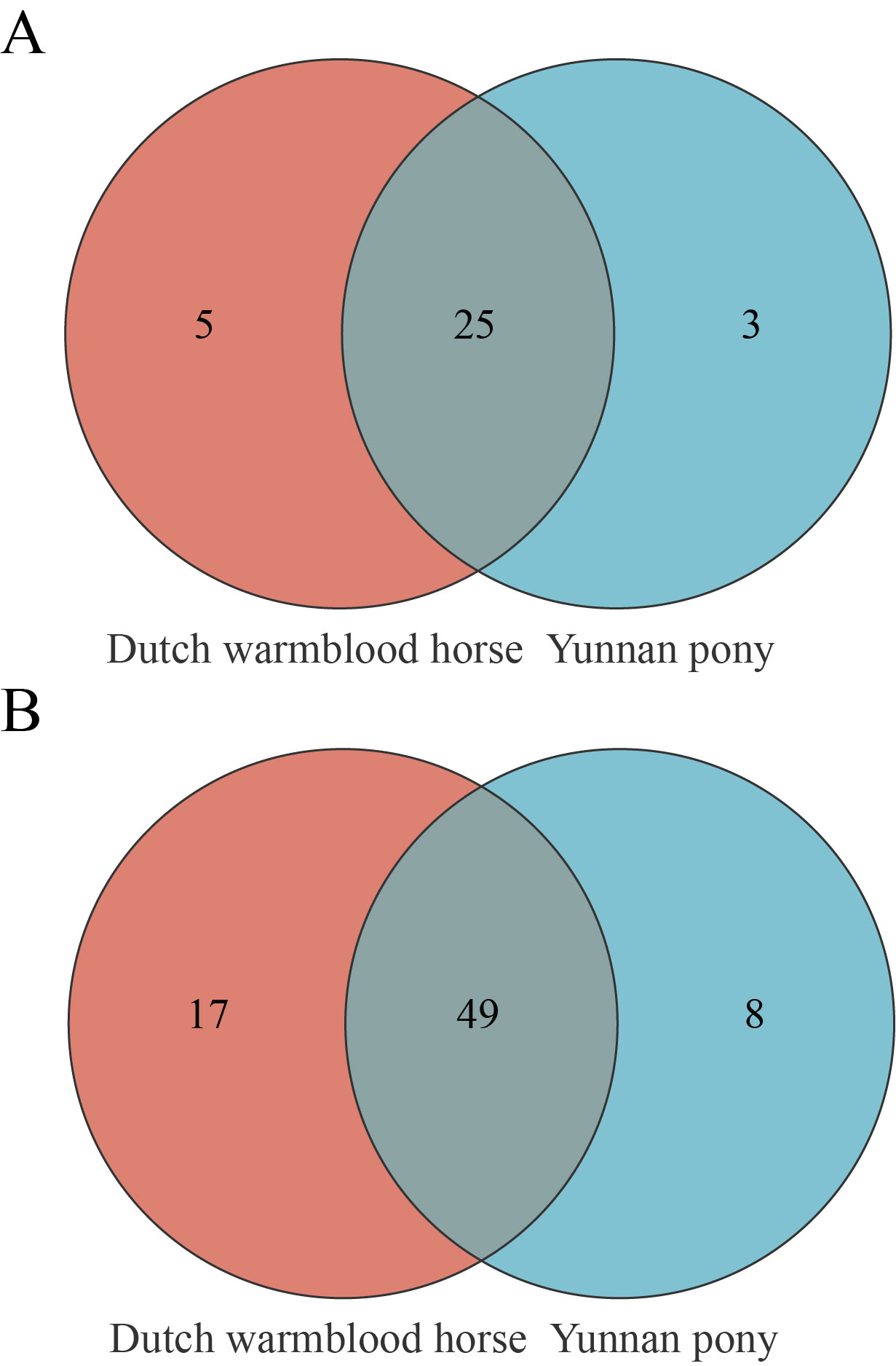

Supplement: Supplementary Figure 1 — Venn diagrams showing shared and unique bacterial genera (A) and ARG subtypes (B) between Yunnan ponies and Dutch Warmblood horses. [file Image_1.jpeg]
